# Complete Genome Assembly of an Auritidibacter ignavus Isolate Obtained from an Ear Infection in Switzerland and a Comparison to Global Isolates

**DOI:** 10.1128/MRA.00291-19

**Published:** 2019-07-11

**Authors:** H. M. B. Seth-Smith, D. Goldenberger, K. A. Bernard, A.-M. Bernier, A. Egli

**Affiliations:** a Division of Clinical Bacteriology and Mycology, University Hospital Basel and University of Basel, Basel, Switzerland; b Applied Microbiology Research, Department of Biomedicine, University of Basel, Basel, Switzerland; c Special Bacteriology Unit, National Microbiology Laboratory, Public Health Agency of Canada, Winnipeg, Manitoba, Canada; d Department of Medical Microbiology, University of Manitoba, Winnipeg, Manitoba, Canada; e Department of Biology, Université de Saint-Boniface, Winnipeg, Manitoba, Canada; University of Maryland School of Medicine

## Abstract

We present the whole-genome sequence of an isolate of Auritidibacter ignavus, associated with ear infections. This complete assembly was compared to genomes of four global isolates, which revealed a high diversity within the species.

## ANNOUNCEMENT

Auritidibacter ignavus, first described in 2011 (1), is a Gram-positive bacillus associated with ear infections and otorrhea. We present the first complete genome assembly to complement the several draft genomes which have been produced (K. A. Bernard, A. L. Pacheco, T. Burdz, D. Wiebe, D. R. Beniac, S. L. Hiebert, T. F. Booth, B. Jakopp, D. Goldenberger, H. M. B. Seth-Smith, A. Egli, and A.-M. Bernier, submitted for publication). Isolate NML 130574 (400516/2013) was obtained from a patient in Basel, Switzerland, with an ear infection in February 2013, described separately (Bernard et al., submitted).

NML 130574 was grown for 48 h on blood agar at 37°C, and DNA was extracted using the Qiagen EZ1 kit and the Qiagen DNeasy blood and tissue kit and sequenced on the Illumina MiSeq platform (300-bp paired ends) after Nextera XT (Illumina) library preparation and the Pacific Biosciences RS II platform using one single-molecule real-time (SMRT) cell at the Functional Genomics Centre in Zurich (FGCZ), Switzerland. Illumina MiSeq reads (949,634 reads; mean coverage, 76×) were quality controlled using FastQC v0.11.8 (http://www.bioinformatics.babraham.ac.uk/projects/fastqc/), hybrid assembled with PacBio reads (76,350 reads; mean coverage, 58×) using Unicycler v0.4.4 (2), resulting in a single scaffold, and annotated using Prokka v1.12 (3), all using default parameters.

The complete genome of Auritidibacter ignavus NML 130574 comprises 2,656,163 bp with 59.5% G+C content. Two rRNA operons were assembled, and there were 48 tRNAs and 2,439 predicted coding sequences (CDSs). One CRISPR with 59 repeat units was identified.

**FIG 1:**
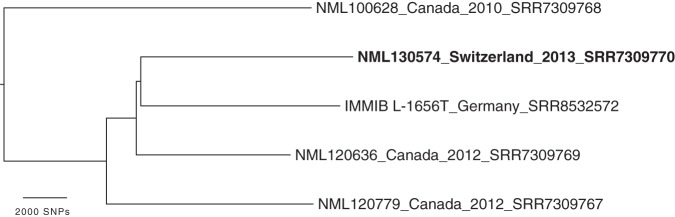
SNP phylogeny of all sequenced isolates of Auritidibacter ignavus. The unrooted phylogeny shows a large diversity between strains. The scale indicates the number of SNPs along branches. The isolate with a complete genome sequence, used here as a reference, is shown in bold.

A phylogenetic comparison of all sequenced isolates of Auritidibacter ignavus was performed in CLC Genomics Workbench v10.1.1, with mapping, variant calling, and single nucleotide polymorphism (SNP) tree creation using parameters that differed from the default as follows: variant calling with 10× minimum coverage, minimum count of 10, and 70% minimum frequency and SNP tree creation with 10× minimum coverage, 10% minimum coverage, 0 prune distance, and including multinucleotide variants (MNVs). The phylogeny shows a diversity of almost 50,000 SNPs across the tree (Fig. 1). The pangenome of this species may also be substantial, as each isolate appears to carry additional genomic regions making up 4 to 12% of the genome (Table 1).

**TABLE 1:** Isolate and mapping details of comparative analysis

Isolate	Source (yr, location)^*a*^	SRA accession no.	Coverage to CP031746 (×) (mean ± SD)	% of CP031746 covered by reads	% reads mapping to CP031746
NML 130574^*b*^	2013, Switzerland	SRR7309770	76 ± 14	100	99
IMMIB L-1656^T^	<2009, Germany^*c*^	SRR8532572	182 ± 75	92	90
NML 100628	2010, Canada	SRR7309768	70 ± 30	90	90
NML 120636	2012, Canada	SRR7309769	89 ± 32	92	96
NML 120779	2012, Canada	SRR7309767	93 ± 34	96	88

a Bernard et al., submitted.

b Self-mapping.

c <, before.

We hope that this complete genome and analysis will inform and aid further research on this pathogen.

### Data availability.

This whole-genome sequencing project has been deposited in GenBank under the accession no. CP031746. The version described in this paper is the first version, CP031746.1. Reads have been deposited under BioProject no. PRJNA437673.
